# Relationship between rumination, self-compassion, and psychological health among Japanese university students: A cross-sectional study

**DOI:** 10.1371/journal.pone.0297691

**Published:** 2024-01-31

**Authors:** Kaho Yamasaki, Ayaka Sampei, Hiromitsu Miyata

**Affiliations:** 1 School of Culture, Media and Society, Waseda University, Shinjuku-ku, Tokyo, Japan; 2 Faculty of Letters, Arts and Sciences, Waseda University, Shinjuku-ku, Tokyo, Japan; Albanian University, ALBANIA

## Abstract

**Objectives:**

Rumination is suggested to exacerbate psychological health, but there is room for investigating the potential protecting factors for individuals with high ruminative tendencies against psychological symptoms. The present cross-sectional study aimed to uncover the associations between rumination as a maladaptive dimension of self-focus, self-compassion, and psychological health, and whether and how components of self-compassion may moderate the associations between rumination and psychological health in Japanese undergraduate students.

**Methods:**

A questionnaire survey was conducted by using both an online platform and a paper-based questionnaire. The survey included established Japanese versions of psychological scales on rumination, self-compassion, perceived stress, trait anxiety, and depression.

**Results:**

The results revealed statistically significant positive correlations between rumination and negative dimensions of self-compassion, and rumination and psychological symptoms. Positive dimensions of self-compassion were significantly negatively correlated with both rumination and psychological symptoms. Reflection as a positive dimension of self-focus did not show significant correlations with most psychological variables. Furthermore, hierarchical multiple regression analyses involving subscales of self-compassion and their interactions with rumination as predictors revealed that the interactions between rumination and components of self-compassion, i.e., over-identification and mindfulness, significantly predicted trait anxiety. Higher rumination predicted higher anxiety when over-identification was high, but not when over-identification was low. Also, lower rumination predicted lower anxiety when mindfulness was high, but not when mindfulness was low.

**Conclusions:**

The results suggest significant associations between rumination, components of self-compassion, and psychological health in a population of Japanese undergraduate students. The data also suggest that components of self-compassion play moderating roles in the relationship between rumination and psychological health, potentially serving as aggravating/protective factors for psychological health. Longitudinal studies and comparisons between different cultures should be conducted in the future quest.

## Introduction

Self-focus refers to a psychological state of paying attention to the inner-self, as well as a personality trait that is likely to lead to such psychological state [1]. Levels of self-focus are suggested to be associated with symptoms of psychopathology such as perceived stress, trait anxiety, and depression (hereinafter referred to as “psychological symptoms”) [2, 3]. A growing amount of recent evidence suggests that self-focus involves maladaptive as well as adaptive aspects [4, 5]. Trapnell and Campbell [5] made a distinction between rumination and reflection, each of which reflects a maladaptive and adaptive dimension of self-focus. Rumination is considered to be a negative, repetitive, chronic, and highly sustainable thinking pattern motivated by perceived threats, losses, and injustices to oneself. Reflection, by contrast, is deemed as a psychological trait characterized by a propensity to direct attention to the inner self motivated by intellectual curiosity. In relation to the Big Five personality traits, Trapnell and Campbell [5] suggested that rumination is positively associated with neuroticism, whereas reflection is positively associated with openness. Rumination can be distinguished from relevant cognitive processes such as worry, which is a maladaptive repetitive thinking associated with anxiety disorder. Specifically, worry is deemed to involve anticipating future threats and aiming to avoid negative emotions and images. Rumination is considered to entail focusing on negative aspects of oneself and attempting to derive meanings from past events [6].

Maladaptive functions of rumination have been supported by a growing amount of theory and evidence that suggests its positive relations to psychological symptoms (for a review, see [7]), while both aspects of self-focus are suggested to be significantly associated with psychological health [5, 7]. Beck and Haigh [8] proposed a cognitive model on psychopathology assuming that internal events such as rumination can initiate and maintain psychological symptoms by activating maladaptive cognitive schemas and triggering biased beliefs, focus, and maladaptive behavior. In accordance with these ideas, several meta-analyses have suggested that rumination can lead to increased levels of anxiety and depression [7–9]. In an empirical study, Balsamo, Carlucci, Sergi, Murdock, and Saggino [10] reported that co-rumination with friends were positively related to depression mediated by activation of maladaptive cognitive schemas. Balsamo [11] also reported that anger showed a positive association with depression mediated by increased rumination. Michl, McLaughlin, Shepherd, and Nolen-Hoeksema [12] demonstrated that perceived stress was positively associated with symptoms of depression or anxiety via increased rumination. Cook, Mostazir, and Watkins [13] reported that a rumination-focused cognitive behavior therapy intervention involving undergraduate students with relatively high levels of rumination resulted in reduced levels of perceived stress and depression. Among Japanese undergraduate students, previous studies on rumination/reflection have investigated their relations to depression, and have concurred that rumination positively predicts depression, whereas reflection negatively impacts depression [14–16]. Yamakoshi and Tsuchiya [17] reported that rumination negatively influenced stress responses such as anxiety and depression as well as psychological well-being, whereas reflection had positive impacts on these variables in Japanese university athletes. Thus, among Japanese undergraduates, individuals with a high level of rumination are prone to having mental health problems, whereas those with a high level of reflection are likely to maintain good psychological health [14–17].

Given these literature, it should be necessary to determine potential factors that could contribute to protecting psychological health of individuals with a high ruminating tendency [5, 7–17]. The present study focuses on the concept of self-compassion, which represents a mental attitude of directing kindness toward oneself, nonjudgmentally accepting experience at the present moment (mindfulness), and recognizing that such experience is shared by others when experiencing pain and worry [18–20]. Previous studies have shown positive associations between self-compassion and desirable psychological outcomes. Individuals with higher dispositional self-compassion have been reported to be less prone to have negative thoughts and experience negative affections than are those with lower self-compassion [21–23]. Dispositional self-compassion has also been positively associated with higher well-being [24], and negatively associated with perceived stress, anxiety, and depression [25, 26]. Preceding studies have also suggested that self-compassion shows robust buffering effects on psychological symptoms. In a recent study, Beshai, Salimuddin, Rafaie, and Maierhoffer [27] demonstrated that dispositional self-compassion as well as mindfulness significantly moderated the effects of stress relevant to COVID-19 pandemic on depression and anxiety (see also [28, 29]).

Constructs and factor structures of self-compassion have been studied over the two decades. Neff [19] developed the original Self-Compassion Scale (SCS) and proposed that self-compassion involves three positive components, i.e., *self-kindness*, *common humanity*, and *mindfulness*, as well as three negative components i.e., *self-judgment*, *isolation*, and *over-identification*, as constructs opposite to each positive component. Self-kindness denotes a tendency of being gentle, supportive, and understanding toward oneself without judging oneself for personal shortcomings (self-judgment). Common humanity represents a tendency to recognize a shared human experience with the understanding that all humans fail and make mistakes and that all people lead imperfect lives, instead of experiencing isolation because of one’s imperfection, egocentrically feeling that s/he is the only one who has failed or is suffering (isolation). Mindfulness refers to being aware of one’s moment-to-moment experience of suffering with clarity and balance, without being caught up in an exaggerated storyline about negative aspects of oneself or one’s life experience (over-identification; see also [30]). Recent studies examining the factor structure of the SCS have indicated that these components of self-compassion can be divided into two distinct constructs, i.e., *self-compassion* and *self-coldness*, each of which denotes to the positive and negative dimension of self-compassion [31]. Empirical data to date have suggested that these two dimensions of self-compassion may be differentially associated with psychological health. Brenner, Heath, Vogel, and Credé [31] showed that self-coldness significantly predicts both psychological well-being and psychological symptoms, whereas self-compassion predicts only psychological well-being. Muris and Petrocchi [32] conducted a meta-analysis of the literature and reported that negative dimensions of self-compassion were more strongly linked to mental health problems than its positive dimensions. In the Japanese population, Arimitsu [18] developed and validated a Japanese version of the SCS by involving undergraduate students. A six-factor model that involves all subscales of self-compassion showed a better fit to the data than the one-factor model, the higher one-factor model involving the total scores of six subscales, or the higher two-factor model involving the total scores of positive and negative dimensions of self-compassion. Arimitsu [18] therefore suggested a six-factor structure for this Japanese version of the scale. Based on Arimitsu [18], it seems reasonable to confirm the six-factor structure of self-compassion when involving a Japanese population.

Moderating roles of each component of self-compassion on psychological health have been suggested by several recent empirical studies. Wong and Mak [33] investigated whether and how each three positive component of self-compassion differentially moderated associations between cognitive traits related to interpersonal relationships including sociotropy, autonomy, and self-criticism, and depression among Chinese adults in Hong Kong. Results suggested that both self-kindness and mindfulness moderated the associations between autonomy and depression, and between self-criticism and depression. Common humanity was also suggested to moderate the association between self-criticism and depression. Wong and Mak [33] noted the significance of differentially investigating moderating roles of each component of self-compassion, because these components are conceptually distinct and are experienced differently (see also [20, 34]). More recently, Browne, Duarte, Miller, Schwartz, and LoPresti [29] reported that, among college students in America who face discrimination, positive associations between experience of racial discrimination and anxiety/somatic symptoms were stronger when self-judgment was high than when it was low. These data seem to support the idea that it is reasonable to examine the moderating effect of each six component of self-compassion, assuming that a six-factor structure is confirmed for the studied sample.

With regard to rumination and psychological health, Brown, Hughes, Campbell, and Cherry [35] reported that self-compassion predicts lower anxiety and depression, and that these associations are mediated by rumination and worry. Interventions involving compassion-focused treatment have also been shown to be effective for enhancing levels of self-compassion and reducing levels of rumination and psychological symptoms [36]. However, whether and how each component of self-compassion may play moderating roles in the relationship between rumination and psychological symptoms has not been extensively examined. To date, Samaie and Farahani [37] reported that a significant interaction between rumination and self-compassion predicted perceived stress in undergraduate students in Iran; however, they did not uncover how self-compassion and/or its components may play moderating roles in further detail. It seems reasonable to assume that each component of self-compassion buffer the associations between rumination and psychological symptoms. Regarding self-kindness, individuals with high ruminative tendencies are considered to persist in and negatively judge their failures or shortcomings, which may lead to worse psychological symptoms [6]. Self-kindness may allow individuals who tend to ruminate about their inadequacy or weakness to be kinder to themselves when faced with difficulties [33]. Thus, self-kindness may well protect such individuals against psychological symptoms. Regarding common humanity, given that rumination represents a repetitive thinking pattern toward one’s inner feelings, thoughts, and sensations [5], individuals with high ruminative tendencies may be prone to feel isolated when faced with difficulties or failures. Common humanity is considered to help such individuals to appreciate failures as common to all human beings and reduce frustration [33]. Thus, common humanity may protect individuals with high dispositional rumination from worse psychological symptoms. Regarding mindfulness, individuals with high ruminative tendencies are considered to have negative views or impressions against facts or experiences (i.e., minimizing their successes and/or maximizing their suffering), which may impede good problem-solving and exacerbate psychological health [6]. Mindfulness can enable individuals to take a balanced and broad view of the situation and to generate alternative solutions, without being preoccupied with pain and failure [33]. Thus, mindfulness is expected to serve as a protective factor against worse psychological symptoms resulting from rumination. In contrast, each negative component of self-compassion, i.e., self-judgment, isolation, and over-identification, can be assumed to boost the aggravating effects of rumination on psychological symptoms, in ways opposite to the positive components of self-compassion.

Within these research contexts, the present study aimed to uncover (a) the associations between rumination/reflection as maladaptive/adaptive dimension of self-focus, self-compassion, and psychological health, as well as (b) potential moderating roles of the components of self-compassion in the associations between rumination and psychological health by involving Japanese undergraduate students. Based on the abovementioned preceding studies, we hypothesized that (1) rumination, although not reflection, is negatively associated with self-compassion and positively associated with psychological symptoms, and that (2) each component of self-compassion may moderate the associations between rumination and psychological symptoms. Given the suggested maladaptive functions of rumination, we expected that ruminative tendencies would show positive correlations with negative components of self-compassion, negative correlations with positive components of self-compassion, and positive correlations with perceived stress, anxiety, and depression. By contrast, based on the suggested adaptive functions of reflection, we expected that reflection shows correlations opposite to rumination. Also, given existing literature suggesting that components of self-compassion may serve as protective/aggravating factors for psychological health, we expected that rumination would less likely predict higher anxiety and depression when self-kindness, common humanity, and/or mindfulness were high compared with when they were low. In contrast, negative components of self-compassion were expected to show moderating effects in ways opposite to the positive components.

## Methods

### Participants and procedures

A total of 340 healthy Japanese undergraduate students (248 females and 86 males; age range 18–25 years; mean age 19.6 years, *SD* 1.34) participated in the study. Six participants either did not report sex or reported that they were neither female nor male. Another 80 individuals who did not answer any of the psychological scales were excluded. Due to the COVID-19 pandemic, data collection was conducted in a hybrid form, i.e., by using both an online survey and a paper-based questionnaire in parallel. For both data collection methods, a convenience sampling approach was used with more than 2,000 students contacted in total. Due to practical constraints including a relatively low rate of participation and time constraints, it was not feasible to use random sampling methods to better examine fit indices for the factor structure of the SCS [38]. Specifically, 258 participants (192 females and 64 males; sex unknown for two individuals; mean age 19.5 years, *SD* 1.41) completed the online survey by accessing the platform from a link attached to a message sent to the students from the university learning management system (Waseda Moodle). The other 82 participants (56 females and 22 males; sex unknown for four individuals; mean age 19.9 years, *SD* 0.98) completed the paper-based questionnaire. The contents of the survey and instructions were identical in these survey methods. Regarding the data collected online, participants whose time to complete the survey was extremely long or short were excluded from the analysis because either incorrect or uncommon response patterns are suspected for such participants [39, 40]. Specifically, after a log transformation (base e) of the time to complete the survey, nine participants whose time to complete the survey exceeded two *SD*s above or below the mean were excluded. Consequently, data for 331 participants (242 females and 83 males; sex unknown for six individuals; age range 18–25 years; mean age 19.6 years, *SD* 1.34) were included in the analysis. The mean time for these participants to complete the survey was 13.2 minutes (*SD* 12.14).

The platform for the online questionnaire survey was constructed by using a platform designed for various types of online surveys (Qualtrics XM). The paper-based survey was conducted by distributing questionnaire sheets to the students during undergraduate classes. The survey sheets were collected after each 90-minute class, immediately after each participant completed the survey. The participants were informed that the study aimed to assess their normal psychological status and did not intend to evaluate any individuals. The participants were instructed to report their psychological status honestly because there were no “true or false” or “good or bad” answers to each question item. Upon providing informed consent to cooperate, the participants either clicked a checkbox on the platform (online) or provided a written signature (paper-based). The participants then answered the question items and psychological scales by either clicking a checkbox (online) or encircling an option (paper-based) for each question. Data collection was conducted between August 2021 to May 2022. Prior to starting data collection, this study was approved by the Ethics Review Committee on Research with Human Subjects of Waseda University (approval No. 2021–154).

### Psychological scales

The following established Japanese versions of the psychological scales were used.

#### Rumination-reflection

We used the Rumination-Reflection Questionnaire (RRQ; [5]), a commonly used scale to measure the tendency to ruminate about and reflect on oneself. The RRQ is composed of rumination and reflection subscales. Each subscale is composed of 12 statements as items, with each item measured on a 5-point scale ranging from “1 (strongly disagree)” to “5 (strongly agree)”. The present study used the Japanese version of the RRQ developed by Takano and Tanno [15], for which both the rumination and reflection subscales have been confirmed to have good reliability and validity. In the present study, participants were asked about the extent to which each statement matched their normal thinking patterns toward the inner-self. In accordance with the general practice of the RRQ, we separately calculated total scores from the rumination and reflection subscales. The internal consistencies (Cronbach’s α) of the rumination and reflection subscales for the present sample were 0.91 and 0.88, respectively.

#### Self-compassion

The Self-Compassion Scale (SCS; [19]), a widely used scale to assess dispositional levels of self-compassion, was used. The SCS is composed of three core components of self-compassion, i.e., self-kindness (5 items), common humanity (4 items), and mindfulness (4 items), as well as opposite aspects of self-kindness (*self-judgment*; 5 items), common humanity (*isolation*; 4 items), and mindfulness (*over-identification*; 4 items). Each question item involves self-evaluative sentences, and participants are instructed to answer each item by reflecting on their normal status of thoughts, emotions, and behaviors. Each item is rated on a 5-point scale ranging from “1 (almost never)” to “5 (almost always)”. Total SCS scores are calculated after inverting the scores on items from the self-judgment, isolation, and over-identification subscales. Arimitsu [18] developed and validated a Japanese version of the SCS, which was used in the present study. As described above, Arimitsu [18] suggested a six-factor model for this Japanese version. For the present sample, Cronbach’s α for the total SCS was 0.92. Cronbach’s α for each subscale was as follows: 0.87 for self-kindness, 0.76 for common humanity, 0.74 for mindfulness, 0.85 for self-judgment, 0.80 for isolation, and 0.81 for over-identification.

#### Perceived stress

We also used the Perceived Stress Scale (PSS; [41]), which measures comprehensive stress appraisal levels in individuals. The PSS entails perceived and appraised stress and aims to assess the degrees to which a life situation is perceived as stressful. Each item on the PSS concerns whether participants have perceived more requirements than they can cope with, and asks about the extent to which their experience was unpredictable, uncontrollable, and overloading during the previous month. Sumi [42] developed and validated the Japanese version of the PSS, which was used in the present study. The PSS is composed of 14 items, each rated on a 5-point scale ranging from “1 (never)” to “5 (very often)”. Total scores are calculated by summing these items. In the present sample, Cronbach’s α for the total PSS was 0.81.

#### Anxiety

The State-Trait Anxiety Inventory (STAI; [43]), which is one of the most common scales for measuring the extent of anxiety, was used. The STAI makes a distinction between state and trait anxiety, and contains both these components, i.e., STAI-S and STAI-T, respectively. The STAI-S concerns transitory emotional states involving feelings of apprehension and nervousness, whereas the STAI-T measures potential reactivity to stressors, i.e., the extent to which one may enter into an anxious state under stressful conditions. The present study used the STAI-T, for which a Japanese version was developed by Shimizu and Imae [44]. Each component has 20 items that are measured on a 4-point scale ranging from “1 (almost never)” to “4 (almost always)”. For each question item, respondents are required to reflect on their normal levels of anxiety. The total scores from these items were used in the analysis. In the present sample, Cronbach’s α for the total STAI-T was 0.90.

#### Depression

We used the Center for Epidemiologic Studies Depression Scale (CES-D; [45]), which was originally developed for epidemiologic studies of depression. Shima et al. [46] developed a Japanese version of the CES-D and demonstrated its clinical effectiveness for the assessment of depressive symptoms. The CES-D, which is commonly used to self-estimate degrees of depression, is composed of 20 items concerning psychosomatic depressive symptoms related to emotion, appetite, and sleep. For each item, respondents self-report how frequently they have experienced each physical and/or mental status during the past week. Each item is rated on a 4-choice scale from A to D, with each letter corresponding to *rarely or none of the time* (less than 1 day per week), *some or a little of the time* (1–2 days), *occasionally or a moderate amount of time* (3–4 days), and *most or all of the time* (5–7 days), respectively. Each letter is subsequently scored from 0 to 3, respectively. We summed the scores from all items and used the total scores in the analysis. In the present sample, Cronbach’s α for the total CES-D was 0.90.

### Statistical analysis

Statistical analyses were conducted primarily using HAD version 17 software [47]. Jamovi version 2.3.26 software was also used to conduct confirmatory factor analysis. The first part of the statistical analysis concerned our aim of examining associations between rumination/reflection, self-compassion, and psychological health. Initially, (1) confirmatory factor analyses were conducted for the SCS, in order to examine whether a six-factor model, a two-factor model assuming two subscales of positive and negative dimensions of self-compassion, or a one-factor model shows a good fit for the data, and to determine which model was most suitable for the moderation analyses. Cutoff points for the absolute fit indices were: CFI>.950; RMSEA < .060; SRMR < .080 [48]. Larger CFI and smaller RMSEA/SRMR indicate a better fit for the model. Models that met at least one criterion for these fit indices were adopted. We also examined relative fit indices including AIC and BIC, for which both smaller AIC and BIC indicate a better fit for the model. Because a six-factor structure was suggested for the present sample, subsequent analyses were conducted by involving the six subscales of the SCS. In addition, Harman’s single-factor test was conducted for all items from the psychological scales, in order to examine whether a common method bias can be a serious concern for the present data [49]. Next, (2) differences between the two data collection methods, i.e., online or paper-based, were examined for all total/subscale scores from psychological scales and demographic variables, i.e., sex and age, by using either Welch’s *t*-tests (all psychological variables and age) or a Fisher’s exact test (sex). These analyses were conducted in order to confirm whether difference in these platforms resulted in difference in demographic properties of the participants and/or different answering patterns for the psychological scales [50]. After confirming that no statistically significant difference was found between the data collection methods except for age, data from online and paper-based surveys were pooled for subsequent analyses. Then, (3) means and standard deviations (*SD*s) for the total/subscale scores from each psychological scale were calculated. Zero-order correlations (Pearson’s *r*) between total/subscale scores from psychological scales were also calculated to examine associations between these variables.

Furthermore, the second part of the statistical analysis concerned our purpose of examining moderating roles of self-compassion. Specifically, (4) we conducted hierarchical multiple regression analyses in order to examine moderating effects of the six subscales of self-compassion on the relationship between rumination and psychological health. Reflection, which did not show significant correlations with most psychological variables, was not included in these models. Models with the total scores of the STAI-T, and CES-D as dependent variables were examined separately. On the basis of the diathesis-stress model [51–53], we have included the total scores of the PSS as a predictor in these models, instead of assuming perceived stress as a dependent variable. Because we examined interactions in these models, all independent variables were centered for the analyses. In Step 1, demographic variables, i.e., sex and age, and data collection method were included as control variables. Step 2 involved the six subscales the SCS, the rumination subscale from the RRQ, and the total scores from the PSS as predictors to examine their main effects. In Step 3, interactions between the six subscales of the SCS and rumination were included in each model. Following the statistically significant interactions, we further conducted simple slope analyses to uncover the effects of each subscale of the SCS at 1 *SD* above and below the mean. Bonferroni corrected significance levels of α = 0.05/2 were applied for these post-hoc analyses.

## Results

### Confirmatory factor analyses

Regarding the SCS, the six-factor model showed an acceptable fit (*χ*^2^ = 952.711, *p* < .001, CFI = .842, RMSEA = .086, SRMR = .073, AIC = 21855.702, BIC = 22205.572), suggesting a six-factor structure for the SCS in the present sample. Moreover, neither a two-factor model (*χ*^2^ = 1388.430, *p* < .001, CFI = .742, RMSEA = .107, SRMR = .085, AIC = 22263.420, BIC = 22560.623) nor a one-factor model (*χ*^2^ = 1830.777, *p* < .001, CFI = .612, RMSEA = .133, SRMR = .110, AIC = 21968.100, BIC = 22250.254) yielded a sufficient model fit. Therefore, scores from the six subscales of the SCS, but not the summed scores, were used for subsequent analyses. In addition, the model involving all observed variables from the psychological scales did not show an adequate fit (*χ*^2^ = 15804.990, *p* < .001, CFI = .428, RMSEA = .078, SRMR = .101), showing that a common method bias is not a serious concern.

### Differences between data collection methods

Total/subscale scores from no psychological scales significantly differed between the two data collection methods (Welch’s *t*-test; *t* = 0.004–1.414, all *ps*>.159). Regarding demographic variables, distribution of sex, i.e., numbers of males and females, did not significantly differ whether the data was collected online or paper-based (Fisher’s exact test; *p* = .558). Age was significantly higher in the online (mean age 19.7 years) than in the paper-based group (mean age 19.3 years) (Welch’s *t*-test; *t* = 1.989, *p* = .049). Thus, whether the data were collected online or paper-based did not result in statistically significant differences in most psychological or demographic variables, except for a slightly significant difference found for age.

### Scores from psychological scales and correlations

Table 1 shows scores from the psychological scales and the correlations between them. The rumination subscale from the RRQ was significantly negatively correlated with the positive subscales from the SCS, i.e., self-kindness, common humanity, and mindfulness, and significantly positively correlated with its negative subscales, i.e., self-judgment, isolation, and over-identification. Scores for rumination also showed significant positive correlations with the total scores from the PSS, STAI-T, and CES-D. By contrast, the reflection subscale from the RRQ did not show significant associations with the other psychological scales, except for a significant positive correlation with the mindfulness subscale from the SCS. Scores from positive subscales from the SCS showed significant negative correlations with total scores from the PSS, STAI-T, and CES-D, whereas scores from its negative subscales showed significant positive correlations with these outcomes. Total scores from the PSS, STAI, and CES-D showed significant positive correlations with each other (Table 1). Thus, higher rumination was significantly associated with lower self-compassion and worse psychological symptoms. In addition, components of self-compassion and psychological symptoms were significantly associated with each other. By contrast, reflection did not show significant associations with other psychological constructs.

**Table 1:** Scores from psychological scales and zero-order correlations between total/subscale scores.

Psychological scale	M (*SD*)	Correlation coefficient (*r*)									
		1	2	3	4	5	6	7	8	9	10
1. RRQ Rumination	44.06 (8.81)	—									
2. RRQ Reflection	39.39 (9.19)	.288***	—								
3. SCS Self-kindness	14.74 (4.35)	–.315***	.006	—							
4. SCS Self-judgment	17.85 (4.53)	.635***	.046	–.509***	—						
5. SCS Common humanity	11.20 (3.42)	–.257***	.088	.406***	–.375***	—					
6. SCS Isolation	12.41 (3.98)	.567***	.035	–.277***	.646***	–.355***	—				
7. SCS Mindfulness	12.04 (3.08)	–.296***	.173**	.583***	–.400***	.557***	–.378***	—			
8. SCS Over-identification	15.08 (3.52)	.738***	.070	–.309***	.721***	–.338***	.684***	–.412***	—		
9. PSS total	32.02 (8.61)	.539***	–.035	–.376***	.589***	–.354***	.532***	–.370***	.572***	—	
10. STAI-T total	50.95 (10.98)	.675***	.006	–.396***	.694***	–.395***	.672***	–.443***	.726***	.673***	—
11. CES-D total	17.78 (11.05)	.478***	–.001	–.314***	.527***	–.229***	.482***	–.272***	.475***	.679***	.740***

***: *p* <. 001

RRQ: Rumination-Reflection Questionnaire; SCS: Self-Compassion Scale; PSS: Perceived Stress Scale; STAI-T: State-Trait Anxiety Inventory Trait; CES-D: Center for Epidemiologic Studies Depression Scale; M: Mean; SD: Standard deviation.

### Moderating effects of self-compassion

Results from the hierarchical multiple regression analyses are shown in Table 2. The two models involving the STAI-T and CES-D scores as dependent variables both showed a significant increase in the coefficient of determination (*R*^*2*^ values) from Step 1 to Step 2. Increase in the *R*^*2*^ values from Step 2 to Step 3 was significant in the model involving the STAI-T, although not in the model involving the CES-D. In Step 3, rumination, perceived stress, and negative components of self-compassion including isolation and over-identification significantly positively predicted the STAI-T scores (*β* = .188–.238, all *ps* < .003). Moreover, interactions between rumination and over-identification (*β* = .206, *p* = .002), and between rumination and mindfulness (*β* = .107, *p* = .048) both significantly predicted the STAI-T scores. Also, both perceived stress (*β* = .509, *p* < .001) and self-judgment (*β* = .157, *p* = .034) significantly positively predicted the CES-D scores; however, no statistically significant interactions were obtained for the model involving the CES-D. Variance inflation factor (VIF) values for the independent variables in both these models (Step 3) were lower than 4.7 (1.056–4.667 for the model involving the STAI-T and 1.159–4.669 for the model involving the CES-D), indicating the absence of severe multicollinearity.

**Table 2:** Results of hierarchical multiple regression analyses to examine moderating roles of the subscales of self-compassion (*β* coefficients).

Independent Variables	STAI-T			CES-D		
	Step 1	Step 2	Step 3	Step 1	Step 2	Step 3
Step 1						
Sex	.101	.036	.036	–.046	–.051	–.043
Age	.008	.070*	.062	.094	.095*	.095*
Data collection method	.002	.045	.048	–.003	.054	.060
Step 2						
Rumination		.199***	.218***		.090	.128
PSS		.258***	.238***		.519***	.509***
Self-kindness		.004	–.021		–.025	–.019
Self-judgment		.141*	.109		.144*	.157*
Common humanity		–.054	–.061		.047	.047
Isolation		.190***	.201***		.138*	.122
Mindfulness		–.064	–.062		.014	.002
Over-identification		.156*	.188**		–.057	–.054
Step 3						
Rumination × Self-kindness			–.090			–.061
Rumination × Self-judgment			–.133			.109
Rumination × Common humanity humanity			.025			.041
Rumination × Isolation			–.013			.033
Rumination × Mindfulness			.107*			.097
Rumination × Over-identification			.206**			–.009
*R* ^*2*^	.010	.703***	.718***	.012	.517***	.532***
*ΔR* ^*2*^		.693***	.015*		.505***	.015

*: *p* <. 05;

**: *p* <. 01;

***: *p* <. 001

PSS: Perceived Stress Scale; STAI-T: State-Trait Anxiety Inventory Trait; CES-D: Center for Epidemiologic Studies Depression Scale.

**Fig 1:**
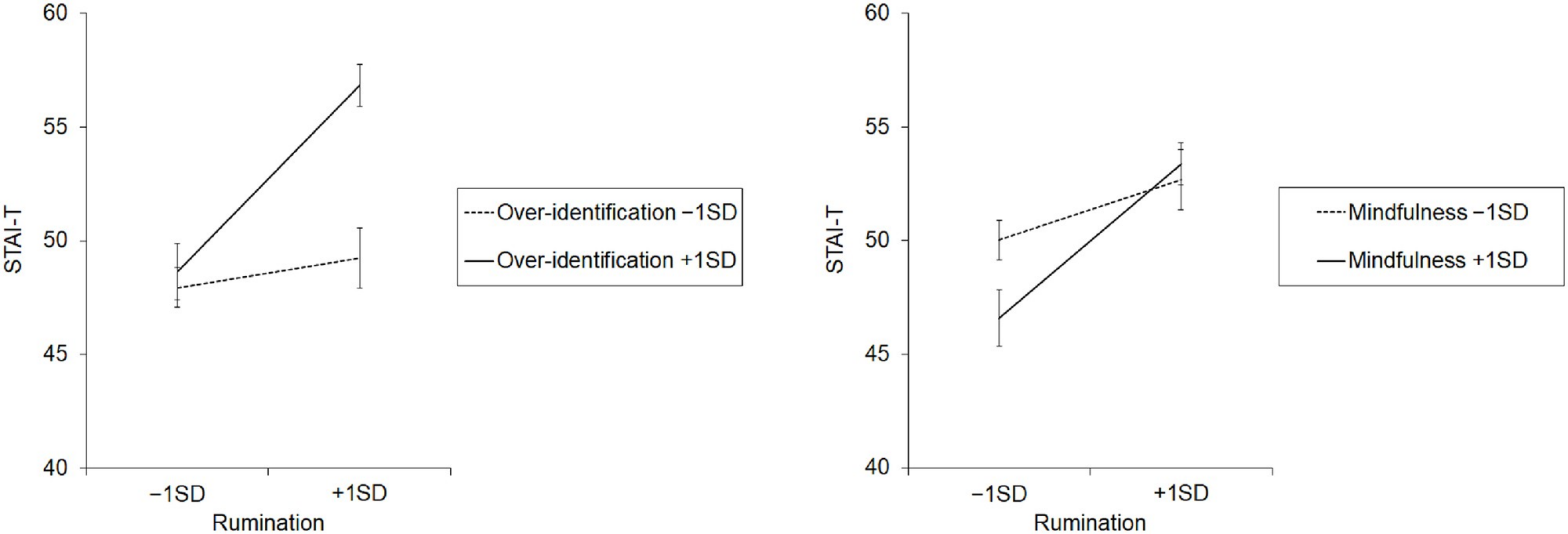
Results of simple slope analyses showing the moderating effects of the subscales of self-compassion. Error bars indicate ±1 standard error. STAI-T: State-Trait Anxiety Inventory Trait; SD: Standard deviation.

Fig 1 shows results from the post-hoc simple slope analyses for the model involving the STAI-T. Higher rumination significantly predicted higher anxiety when over-identification was high (*β* = .375, *p* < .001, corrected), although not when over-identification was low (*β* = .060, *p*>.05, corrected). Thus, ruminative tendency was significantly associated with worse psychological symptoms only in individuals high in over-identification. Also, lower rumination significantly predicted lower anxiety when mindfulness was high (*β* = .312, *p* < .001, corrected), although not when mindfulness was low (*β* = .123, *p*>.05, corrected). Thus, in individuals high in mindfulness, lower ruminative tendency was likely to predict lower trait anxiety. By contrast, in individuals low in mindfulness, anxiety stayed high even with lower rumination.

## Discussion

The purposes of the present study were to examine the associations between rumination/reflection, self-compassion, and psychological health, and whether and how components of dispositional self-compassion may play moderating roles in the relationship between rumination and psychological health in Japanese undergraduate students. The results showed that higher ruminative tendencies were significantly negatively associated with positive components of self-compassion, positively associated with negative components of self-compassion, and positively associated with psychological symptoms. Moreover, the results further suggested that some components of self-compassion moderate the relationships between rumination and trait anxiety.

### Associations between variables

The results from the correlation and multiple regression analyses support our initial hypothesis that higher rumination is negatively associated with self-compassion and is positively associated with worse psychological symptoms. Correlation analysis apparently revealed that ruminative tendencies were negatively correlated with positive dimensions of self-compassion, positively correlated with negative dimensions of self-compassion, and positively correlated with stress/anxiety/depression. Regression analyses further showed that some negative dimensions of self-compassion were significantly associated with worse psychological symptoms, with isolation and over-identification predicting higher anxiety and self-judgment predicting higher depression. These results are consistent with preceding studies including those outlined in the Introduction. For example, Mills, Gilbert, Bellew, McEwan, and Gale [54] suggested that positive components of self-compassion, i.e., self-kindness, common humanity, and mindfulness, were negatively correlated with depression. Michl, McLaughlin, Shepherd, and Nolen-Hoeksema [12] indicated that ruminative tendencies, self-evaluations of stressful life events, and levels of anxiety and depression were positively associated with each other. Cook, Mostazir, and Watkins [13] reported that a rumination-focused psychotherapy intervention alleviated anxiety and depression in undergraduate students with high levels of dispositional rumination and worry (see also [5–9]). Consistent with these studies, the present data support the view that rumination is robustly associated with lower psychological health. Because rumination has been negatively associated with psychological health in the population of Japanese undergraduate students [14–17], the present study seems to add consistent and novel evidence to this literature by demonstrating associations between components of self-compassion and these psychological variables in this population.

By contrast, reflection did not reveal significant correlations with most psychological variables including self-compassion and psychological symptoms, except that reflection was significantly positively correlated with the mindfulness subscale of the SCS. These generally non-significant correlations are not in line with our expectations that reflection would show associations with psychological variables in ways opposite to rumination. Nevertheless, these correlations are consistent with those reported in previous studies involving Japanese undergraduate students. Takano and Tanno [15] found that reflection was significantly positively correlated with openness, although it did not show a significant correlation with depression. Yamakoshi and Tsuchiya [17] reported that reflection was significantly correlated with subscales of subjective well-being, but not with stress response. Because reflection might be more strongly associated with psychological well-being than with psychological symptoms, further investigations by including other scales on psychological health would be required to further uncover these issues.

### Moderating roles of self-compassion

Moderating effects of self-compassion on the associations between rumination and trait anxiety were found for some components of self-compassion, i.e., over-identification and mindfulness. Regarding over-identification, higher rumination predicted higher anxiety when over-identification was high, but not when it was low. In Japanese university/college students, Matsumoto [55] showed that individuals with higher rumination experience stronger discomfort toward having uncomfortable emotions and are devoid of decentering, i.e., separation of thoughts from facts, resulting in worse psychological symptoms. Given that over-identification represents a state where individuals are overwhelmed by and identified with negative emotions and cognitions [19, 30], higher over-identification would be likely to lead to a lack of decentering and further worsen psychological symptoms. The present results seem consistent with these research contexts and suggest that over-identification as a negative dimension of self-compassion can have aggravating effects in the association between rumination and psychological symptoms. Regarding mindfulness, lower rumination predicted lower trait anxiety when mindfulness was high, but not when it was low. These results are consistent with our hypothesis that mindfulness as a component of self-compassion can be a protective factor against psychological symptoms that result from rumination [33]. The results also seem parallel to a good amount of research suggesting that dispositional mindfulness can have buffering effects against psychological symptoms (e.g., [27, 28, 56, 57]). In the present study, mindfulness appeared to contribute to reduced anxiety only when ruminative tendency was low, and not when it was high. This might in part result from the differences in the constructs of mindfulness assumed in other scales on mindfulness such as the Five Facet Mindfulness Questionnaire [58] and mindfulness as a subscale of the SCS, which could further be addressed by introducing other scales on dispositional mindfulness. Given a relatively small amount of research showing moderating effects each component of self-compassion [20, 29, 33, 34], these data should contribute to the literature by providing novel evidence suggesting that some dimensions of self-compassion can protect individuals who tend to engage in maladaptive repetitive thinking patterns against impeding psychological health.

By contrast, self-kindness and common humanity as well as their opposite constructs, i.e., self-judgment and isolation, did not show significant moderating roles in the relationships between rumination and trait anxiety. Also, no components of self-compassion demonstrated moderating effects on the relationships between rumination and depression, even though self-judgment significantly predicted higher depression. Together with the significant moderating effects as described above, these results would support the idea that each component of self-compassion play differential moderating roles in the associations between rumination and psychological symptoms. On the other hand, because a large part of interaction was non-significant, the results did not reveal robust moderating effects of components of self-compassion on psychological symptoms. In fact, previous studies reported moderating effects of self-kindness, self-judgment, common humanity, as well as mindfulness on psychological symptoms [29, 33]. Because components relevant to mindfulness showed significant moderating effects in the present study, one future direction may be to examine whether mindfulness and/or attention control may more apparently buffer the associations between rumination and psychological symptoms than do components of self-compassion. These ideas seem to be supported by previous research that identified distraction and divided attention as cognitive functions that can alleviate impacts of rumination on depression [59, 60].

### Factor structure of self-compassion

Whereas the original Self-Compassion Scale developed by Neff [19] proposed a six-factor model and a higher one-factor model for self-compassion, a large part of more recent literature has suggested a two-factor structure, i.e., self-compassion and self-coldness as positive and negative dimensions of self-compassion, respectively [31, 32]. In contrast to these studies, the Japanese version of SCS developed by Arimitsu [18] suggested a six-factor structure, with relatively weaker correlations between positive and negative components of self-compassion than the original SCS. These results were generally replicated in the present sample involving Japanese undergraduate students. As Arimitsu [18] noted, these differences may potentially be attributable to both issues on translation of question items and cultural backgrounds. To further address these issues, further modifications to the Japanese version of SCS would be desirable so that the scale has a higher one-factor structure, which should provide a rationale for calculating the total SCS scores. It would also be promising to better specify the influence of cultural factors that may underlie these differences. For example, whereas self-judgment is considered to be a negative dimension of self-compassion within the SCS, some Eastern cultures including Japan assume that self-judgment can positively lead to improvement of the self [61]. In the Eastern cultures, Buddhism-based thoughts such as compassion may well influence dispositional self-compassion [18, 30]. Optimal factor structures can thus differ between populations with different cultural backgrounds, which may be better addressed in the future studies.

### Limitations and future perspectives

Despite the novel findings and suggestions, one limitation of the present study concerns the generalizability of the results to different populations. Whereas previous studies have consistently suggested associations between higher ruminative tendencies and psychological health, the magnitude of these associations has been suggested to vary by age and/or cultural background. For example, Ricarte, Ros, Latorre, and Barry [62] reported that young participants in their twenties are more prone to ruminate than are older participants in their seventies. Regarding cultural differences, Tsai, Chang, Sanna, and Herringshaw [63] suggested that European Americans high in ruminative tendencies show lower levels of anxiety and depression when happiness is high, whereas Asian-Americans who tend to ruminate show high anxiety and depression regardless of happiness level. Because the moderating effects of components of self-compassion as shown in the present study have not been demonstrated in other populations, whether such effects may be generalized to wider populations differing in age and/or cultural background should be investigated.

A second limitation relates to the fact that the present study employed a cross-sectional design with data collection conducted at one time stamp. In this design, the time course of the potential changes in psychological traits and their relationships through relatively long periods cannot be uncovered. Intervention studies involving the practice of self-compassion should be further conducted in this context. Mental practices including self-compassion are known to be effective in ameliorating psychological health [64]. For example, a self-compassion-based program combined with basic mindfulness skills that involves loving-kindness mediation has been shown to reduce depression and anxiety and increase psychological well-being and life satisfaction [65]. Therefore, to examine changes in psychological health, future studies should involve not only longitudinal questionnaire surveys, but also interventions involving self-compassion-based practices. In this frontier, Frostadottir and Dorjee [36] conducted a pilot study and found that interventions involving compassion-focused therapy for patients with psychopathy contributed to enhancing levels of self-compassion and reducing levels of rumination and psychological symptoms. In the educational context, Sawyer, Bailey, Green, Sun, and Robinson [66] conducted a psychoeducational intervention called Resilience, Insight, Self-Compassion, and Empowerment (RISE) by involving nurses and reported improvements in self-compassion, resilience, insight, perceived stress, and burnout. Such self-compassion-based interventions may be proposed as prevention strategies against worsening of psychological symptoms that result from a maladaptive aspect of self-focus, such as rumination. Such practices could also be applied in clinical and psychoeducational contexts to alleviate psychosomatic symptoms such as eating disorders, for which ruminative tendencies can serve as an aggravating factor [6, 36]. These approaches could help to effectively apply the present findings on rumination and self-compassion to multiple practical contexts (S1 Dataset).

## Supporting information

S1 Dataset (XLSX)
